# 1437. Short Versus Long-Course Intravenous Antibiotics for Young Infants with Urinary Tract Infection

**DOI:** 10.1093/ofid/ofab466.1629

**Published:** 2021-12-04

**Authors:** Jolie Lawrence, Laure F Pittet, Samar Hikmat, Eloise J Silvester, Vanessa Clifford, Rodney Hunt, Amanda Gwee

**Affiliations:** 1 The Royal Children’s Hospital, Melbourne, Victoria, Parkville, Victoria, Australia; 2 Murdoch Children’s Research Institute, Parkville, Victoria, Australia; 3 The University of Melbourne, Melbourne, Victoria, Parkville, Victoria, Australia; 4 Monash University, Clayton, Victoria, Australia

## Abstract

**Background:**

Shorter courses of intravenous (IV) antibiotics for young infants with urinary tract infection (UTI) have myriad advantages. As practice shifts toward shorter IV treatment course, this study aimed to determine the safety of early IV-to-oral antibiotic switch, and identify risk factors for bacteraemia with UTI.

**Methods:**

Retrospective audit of infants aged ≤90 days with a positive urine culture at a quaternary paediatric hospital over four years (2016-2020). Data were collected from the hospital electronic medical record and laboratory information system. Short-course IV antibiotic duration was defined as < 48 hours for nonbacteraemic UTI and < 7 days for bacteraemic UTI. Multivariate analysis was used to determine patient factors predicting bacteraemia.

**Results:**

Among 427 infants with nonbacteraemic UTI, 257 (60.2%) were treated for < 48 hours. Clinicians prescribed shorter IV courses to infants who were female, aged >30 days, afebrile, and those without bacteraemia or cerebrospinal fluid pleocytosis. Treatment failure (30-day UTI recurrence) occurred in 6/451 (1.3%) infants. All had nonbacteraemic UTI and only one received < 48 hours of IV antibiotics. None had serious complications (bacteraemia, meningitis, death). Follow-up audiology was performed in 21/31 (68%) infants with cerebrospinal fluid pleocytosis, and one had sensorineural hearing loss. Bacteraemia occurred in 24/451 (5.3%) infants, with 10 receiving < 7 days IV antibiotics with no treatment failure, meningitis or death. Fever and pyelonephritis were independent predictors of bacteraemia.

**Conclusion:**

Short course IV antibiotics for < 48 hours for young infants with nonbacteraemic UTI are safe provided bacterial meningitis has been excluded. Treatment failure and serious complications were rare in young infants with UTI.

**Disclosures:**

**All Authors**: No reported disclosures

